# Development and Validation of a Simple and Reliable HPLC-UV Method for Determining Gemcitabine Levels: Application in Pharmacokinetic Analysis

**DOI:** 10.3390/medicina60060864

**Published:** 2024-05-25

**Authors:** Konstantinos Lafazanis, Elias Begas, Irida Papapostolou, Hermis Iatrou, Nikos Sakellaridis, Dimitrios Vlassopoulos, Konstantinos Dimas

**Affiliations:** 1 Department of Pharmacology, Faculty of Medicine, University of Thessaly, 41500 Larissa, Greece; kostaslfz@gmail.com (K.L.); begas@uth.gr (E.B.); irida.papapostolou@unibe.ch (I.P.); nsakella@uth.gr (N.S.); 2 Industrial Chemistry Laboratory, Department of Chemistry, National and Kapodistrian University of Athens, 10676 Athens, Greece; iatrou@chem.uoa.gr; 3 FORTH, Institute for Electronic Structure and Laser, 71110 Heraklion, Greece; dvlasso@iesl.forth.gr; 4 Department of Materials Science and Technology, University of Crete, 71003 Heraklion, Greece

**Keywords:** gemcitabine, HPLC-UV, pharmacokinetic analysis

## Abstract

*Background and Objectives*: Gemcitabine has been used to treat various solid cancers, including, since 1997, metastatic pancreatic cancer. Here, we developed an HPLC-UV method to determine serum gemcitabine levels and use it in pharmacokinetic studies. *Materials and Methods*: The analysis was performed after a single protein precipitation step on a reversed-phase column, isocratically eluted with sodium phosphate buffer and methanol. For the pharmacokinetic study, NOD/SCID mice received a single dose of gemcitabine at 100 mg/kg by either subcutaneous (SC) or intraperitoneal (IP) administration. Blood samples were collected at 5, 15, and 30 min and 1, 2, 4, and 6 h after the administration of gemcitabine for further analysis. *Results*: The duration of the analysis was ~12.5 min. The calibration curve was linear (r^2^ = 0.999) over the range of 1–400 μM. The mean recovery of GEM was 96.53% and the limit of detection was 0.166 μΜ. T_1/2_, Tmax, Cmax, AUC_0–t_, and clearance were 64.49 min, 5.00 min, 264.88 μmol/L, 9351.95 μmol/L*min, and 0.0103(mg)/(μmol/L)/min, respectively, for the SC administration. The corresponding values for the IP administration were 59.34 min, 5.00 min, 300.73 μmol/L, 8981.35 μmol/L*min and 0.0108(mg)/(μmol/L)/min (not statistically different from the SC administration). *Conclusions*: A simple, valid, sensitive, and inexpensive method for the measurement of gemcitabine in serum has been developed. This method may be useful for monitoring gemcitabine levels in cancer patients as part of therapeutic drug monitoring.

## 1. Introduction

Gemcitabine (GEM, 2′,2′-difluorodeoxycytidine, dFdC) is an extremely important pyrimidine nucleoside and a cytidine analogue that is used in combination chemotherapy for the treatment of non-small cell lung cancer, bladder cancer, and breast cancer, as well as in the treatment of other tumors, such as ovarian cancer, and head and neck cancers [1]. One of the most significant clinical applications of this drug, though, is its use as a first-line treatment as monotherapy in patients with metastatic pancreatic cancer since 1997 [2]. More recently, it has been reported that GEM also inhibits the replication of orthohepevirus A, which causes hepatitis E, by enhancing the signaling of interferon [3].

Following intravenous administration, gemcitabine undergoes extensive metabolism by plasma and liver cytidine deaminase (CDA) to form 2′,2′-difluorodeoxyuridine (dFDU), a compound that has been characterized as the major but inactive metabolite of gemcitabine [4]. GEM enters the cells through cell membrane nucleoside transporters, with the most common transporters being SLC29A1, SLC28A1, and SLC28A3 [1].

Intracellularly, the drug undergoes further modifications by tumor cell intracellular kinases. After entering the cell, gemcitabine is first modified by deoxycytidine kinase (DCK) to gemcitabine monophosphate (dFdCMP), which is the rate-determining step. Two more phosphates are added by other enzymes to form gemcitabine diphosphate (dFdCDP) and finally the pharmacologically active form of the drug, which is gemcitabine triphosphate (dFdCTP). The latter, as deoxycytidine triphosphate, is incorporated into the newly synthesized DNA strands, thus arresting the cellular replication. Although dFdCTP is considered the main pharmacologically active form of the drug, gemcitabine diphosphate is active as well, as it inhibits ribonucleotide reductase (RNR), also known as ribonucleoside diphosphate reductase (rNDP), which is the enzyme that catalyzes the formation of deoxyribonucleotides from ribonucleotides, thus resulting in a substantial decrease in the intracellular pool of nucleotides. The limited intracellular availability of nucleotides drives the cell to increase the uptake of components needed to build nucleotides from outside the cell, which results in a concomitant increase in gemcitabine uptake [1].

In this study, we report the development of a simple, sensitive, inexpensive, and validated HPLC method coupled with UV detection for the quantification of GEM in serum samples that could be further used for TDM purposes in human plasma or serum. The method allowed the unequivocal detection of both GEM and dFDU in both mouse and human serum and was implemented in a pharmacokinetic study of GEM in mice. The results suggest that the developed method may indeed be a useful tool in clinical practice for the therapeutic drug monitoring of GEM administered to cancer patients.

## 2. Materials and Methods

### 2.1. Chemicals and Reagents

Gemcitabine was purchased from TCI (Zwijindrecht, Belgium). 1,7-dimethyluric acid (1,7 U) was purchased from Cayman Chemical (Ann Arbor, MI, USA) and was used as an internal standard (IS). HPLC-grade methanol and acetonitrile were purchased from Chem-Lab (Zedelgem, Belgium) and were used without further purification procedures. Tetrahydrouridine (THU) was purchased from Cayman Chemical Company (Cayman Chemical, Ann Arbor, MI, USA). 2′,2′-difluorodeoxyuridine (dFDU) was purchased from Sigma (Steinheim, Germany).

### 2.2. Instrumental Configuration and Chromatographic Conditions

The instrumentation for the HPLC analysis consisted of a SYKAM S1125 pump system (version 1.0, Eresing, Germany) equipped with an online degasser (ERC Inc., Kawaguchi City, Japan), a FASMA500 UV-vis detector (Rigas Labs, Thessaloniki, Greece) and a Spherisorb S5ODS2 column (4.6 mm × 25 cm) (Milford, MA 01757, USA). The mobile phase consisted of 50 mM sodium phosphate buffer (pH 6.6)—methanol 97:3 (*v*/*v*) and was filtered using 0.2 μM pore-size mixed cellulose ester (MCE) filters (Merck KGaA, Darmstadt, Germany) before use. The separation of GEM, IS, and dFDU was performed using isocratic elution mode. The flow rate was 1.0 mL/min. The detection wavelength was set at 267 nm, and the column was preheated and maintained at 40 °C throughout the analysis using an oven (Lab Alliance, State College, PA, USA).

### 2.3. Preparation of Standard Solutions

The stock solutions of GEM (5 mM) and IS (750 μΜ) were prepared in methanol and stored at −20 °C until use. The working solutions of 10, 100, 200, 500, 1000, 2000, and 4000 μΜ were prepared by diluting the stock solution with ultrapure water and again stored at −20 °C until use. A standard solution of dFDU in ultrapure water was prepared at a concentration of 100 μΜ and stored at −20 °C until further use.

### 2.4. Sample Preparation

The spiked calibration and quality control samples were prepared by adding 20 μL of the respective working solutions of the GEM to 180 μL of blank mouse or human serum. Subsequently, 20 μL of IS stock solution was added to the spiked samples. The samples were then vortex-mixed for one minute. Protein precipitation was achieved by adding 80 μL of 6% *v*/*v* perchloric acid dropwise to the samples. The samples were again vortex-mixed for one minute and incubated on ice for 5 min. At the end of the 5 min incubation, the samples were centrifuged at 13,000 rpm for 13 min at 4 °C. The supernatant from each centrifuged sample was finally collected in a new Eppendorf tube, and 50 μL of the collected supernatant was injected into the chromatographic column for analysis to follow. To assess the applicability of the method in humans, blank and spiked serum samples were prepared using commercially available human serum (Bio-Rad, Athens, Greece) following the same procedure described above.

### 2.5. Method Validation

The method was evaluated for selectivity, the linearity of the detector, response, accuracy, precision, sensitivity, recovery, stability, and carry-over of GEM in serum according to the procedures and guidelines reported by the EMA [5].

The selectivity of the method was determined by analyzing pooled sera extracts obtained from 6 different mice.

The calibration curve was generated using six concentration points. Matrix calibrators were prepared by spiking blank mouse serum with GEM at concentrations of 1, 10, 50, 100, 200, and 400 μM. A linear regression model was used, and linearity was confirmed by regression coefficient (r^2^) values.

To estimate the intra-day and inter-day precision and accuracy of the method, we prepared four quality control (QC) samples by spiking mouse sera to prepare samples with the following concentrations: 3 μΜ, 25 μM, 125 μΜ and 375 μΜ of GEM. For the intra-day precision estimation, five replicates of each QC were prepared and analyzed to determine the accuracy of the method. For the inter-day evaluation, each QC sample was prepared and analyzed daily for five consecutive days. Accuracy was defined by the formula {100 × [(measured–target) concentration/target concentration]}. The imprecision was determined by assessing the percentage coefficient of variance (CV%).

The absolute recovery of the method was estimated by a comparison of the peak areas, which were acquired using freshly prepared extracts of mouse sera spiked with various concentrations of GEM (25, 125, and 375 μΜ) with those that were generated by direct injections of aqueous standard solutions of the same concentrations into the HPLC column.

Sensitivity was determined in accordance with the EMA guidelines as the lowest concentration of the analyte in a sample that can be measured reliably with acceptable accuracy and precision (lower limit of quantification, LLOQ). Moreover, the limit of detection (LOD) was determined as the amount of the extracted analyte with a signal-to-noise (S/N) ratio equal to 3 [6].

The stability of GEM in the mouse serum samples was estimated using two QC samples, one at 25 μM and one at 375 μM, following 8 weeks of storage at −20 °C. The concentrations were recalculated on the same day using a newly constructed calibration curve.

Carry-over was determined by analyzing blank mouse sera extracts after the injection of calibration standards at the upper limit of quantification (ULOQ).

### 2.6. Pharmacokinetic Study

Male immunodeficient mice of the NOD.Cg-Prkdc^SCid^/J strain, 10–12 weeks old, were used for the pharmacokinetic (PK) study. During the experiment, the animals were maintained under specific pathogen-free conditions in a standard temperature (21 °C) and humidity (50%) environment with a 12 h light cycle (7:00 am–7:00 pm) and ad libitum access to food and water. The average body weight of the mice was 27 ± 1 g. GEM was administered either intraperitoneally (IP) or subcutaneously (SC). For each GEM administration route, 40 animals were weighed and randomly divided into 8 groups (5 animals/group). One group was used as a control group (mice did not receive any treatment), and the other 7 groups (1 group of 5 animals for every time point) received a single dose of GEM (100 mg/kg) via either IP or SC administration. Thus, the total number of mice for IP and SC administration routes was 80. Blood samples were collected using the tail vein sampling method at the following time points: 5, 15, and 30 min and 1, 2, 4, and 6 h. The samples were then centrifuged, and the sera were collected. In the collected sera, THU, from a stock concentration of 10 mg/mL in water, was added (1 μL/50 μL serum) as a preservative to avoid GEM degradation [7], and finally, the samples were stored at −80 °C until analysis. For further analysis, samples were processed as described above.

### 2.7. Data Acquisition and Statistical Analysis

For data collection and sample analysis, the Clarity v.8.3.01.131 software (DataApex, Prague, Czech Republic) was used. SPSS (version 26, IBM, Athens, Greece) was used for statistical analysis. The pharmacokinetic analysis was performed using PKSolver add-in [8] for the MS-Excel software (Microsoft^®^ Excel^®^ for Microsoft 365 MSO). Results are expressed as means ± SD. The pharmacokinetic parameters for the two routes of gemcitabine administration were compared using the Mann–Whitney *U* test. Differences were considered significant when the *p*-value was <0.05.

## 3. Results

### 3.1. Method Validation

#### 3.1.1. Selectivity

**Figure 1:**
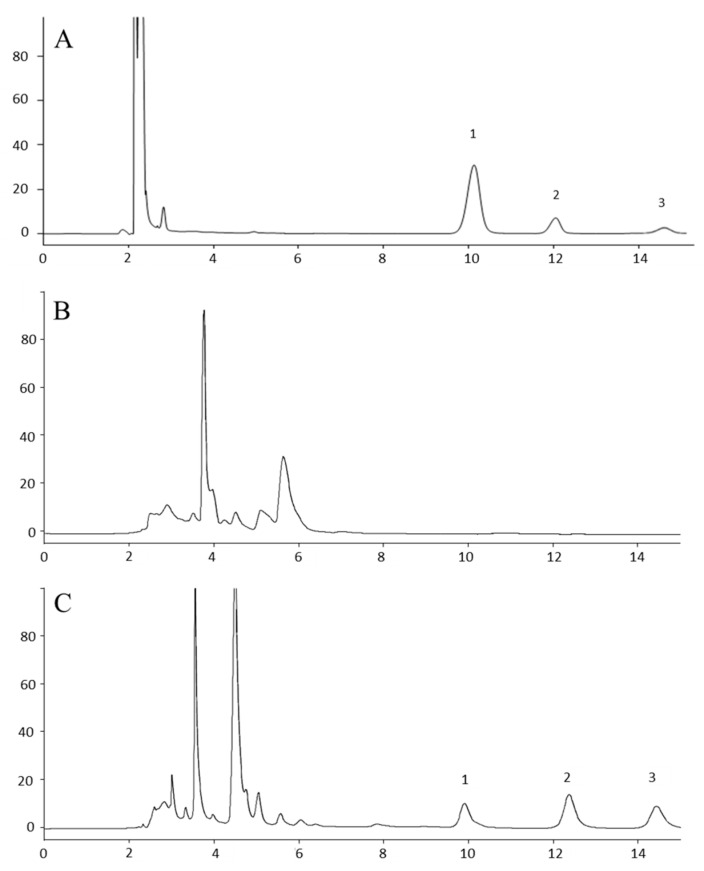

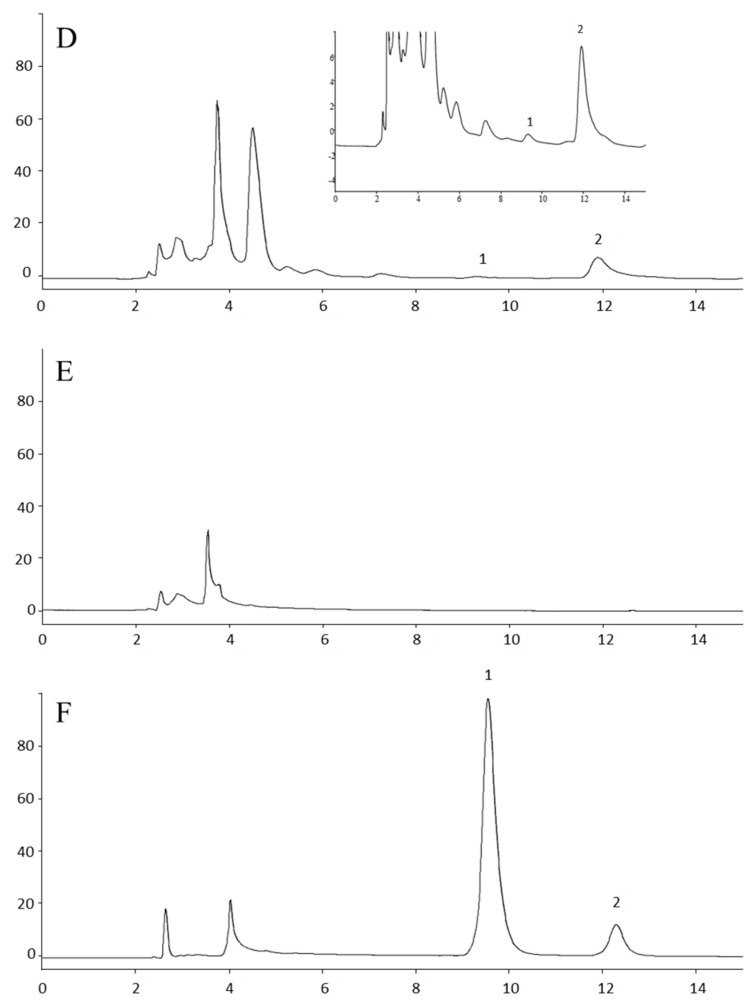
Representative HPLC chromatograms. (**A**) Standard solution of GEM, IS, and dFDU (**B**) Blank mouse serum. (**C**) Representative sample of mouse serum 1 h after SC administration of gemcitabine. (**D**) Lower limit of quantitation (LLOQ) prepared by spiking 1 μΜ gemcitabine and IS in mouse serum. The insert shows a magnification of the LLOQ. (**E**) Blank human serum. (**F**) Human serum spiked with 200 μM of gemcitabine and IS (estimated concentration 209.1 μΜ). 1: gem; 2: IS; 3: dFDU; IS: internal standard. For running conditions, see Materials and Methods. The mean S/N ratio equals 17.48. Noise estimated by the Clarity v.8.3.01.131 software at baseline areas adjacent to the GEM peak.

No serum matrix peaks were observed at the elution time of either the GEM or the IS. A representative chromatogram of extracted pooled blank mouse serum is presented in Figure 1B.

#### 3.1.2. Calibration Curve

The calibration curve followed the equation y = (0.040 ± 0.001) × + 0.011(±0.078) and was found to be linear from 1 to 400 μM (regression coefficient (r^2^), 0.999). No significant difference in its intercept from zero was observed (*p* = 0.886). The concentration of the calibrators was subsequently recalculated according to the EMA guidelines on bioanalytical method validation [8] using the equation mentioned above; the variance (CV%) and bias did not exceed 2.91% and 6.43%, respectively (Table 1). Importantly, the results on CV% and bias did not exceed those recommended by the EMA guidelines.

#### 3.1.3. Recovery

The recovery of GEM was found to be 96.53 ± 6.75%, 93.60 ± 4.62%, and 96.29 ± 6.77%, for the 25 μM, 125 μM and 375 μΜ concentrations, respectively (*n* = 5 for all concentrations). The mean recovery of IS was determined to be 82.77 ± 2.69% (*n* = 27).

#### 3.1.4. Precision and Accuracy

The intra-day (*n* = 5) and inter-day (*n* = 5) results of the method validation are shown in Table 2. The highest bias and CV% for the QC samples used herein were found to be 16.55% and 9.56%, respectively.

#### 3.1.5. Determination of the Lower Limit of Quantitation (LLOQ) and the Limit of Detection (LOD)

The LLOQ of our method was determined as the lowest point of the calibration curve (1.0 μΜ) with a CV of 0.31% and a bias of 6.43% (*n* = 2), which are within the range of the ±20% deviation recommended by the EMA for the LLOQ [8]. The limit of detection, which is the amount of the extracted analyte with a signal-to-noise (S/N) ratio equal to 3 [6], was determined at 0.166 ± 0.022 μΜ (*n* = 3).

#### 3.1.6. Stability

Mean (±SD) measured concentrations of the low-QC and high-QC samples were 22.58 (±2.16) and 390.71 (±47.99), while biases were −9.7 and 4.2%, respectively (*n* = 3). These values are ≤±15%, indicating that GEM remains stable in mice sera stored at −20 °C for the aforementioned period [8].

#### 3.1.7. Carry-Over

Carry-over in the blank samples, following the highest calibration standard, should not be greater than 20% (i.e., 120%) of the analyte response at the LLOQ and 5% of the response for the IS [5]. Mean carry-over (±SD) response (peak area) of GEM in the blank mouse serum samples compared to that of the 400 μΜ calibration standard was 37.68 ± 2.95% (*n* = 3). Mean carry-over response (±SD) of the IS was 0.25 ± 0.10% (*n* = 3).

### 3.2. Pharmacokinetics

**Figure 2:**
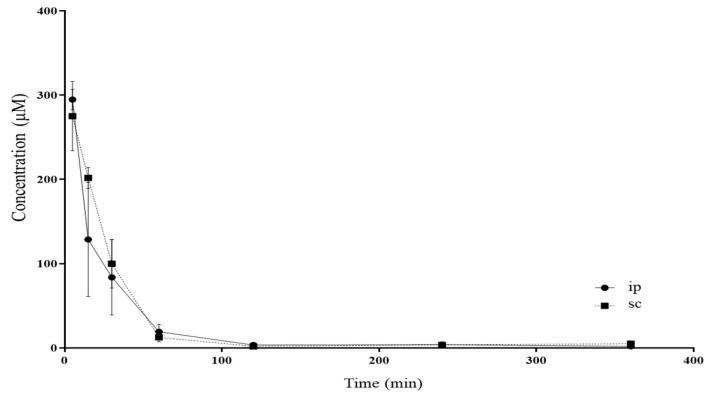
The pharmacokinetic profile of gemcitabine administered in immunodeficient male mice. Gemcitabine was administered as a single dose at 100 mg/kg either IP or SC. Points show average ± SD, *n* = 5.

The method was further applied to the pharmacokinetic analysis of GEM in immunodeficient mice of the NOD.Cg-Prkdc^scid^/J strain following SC and IP administration of the drug. This is an immunocompromised strain that is very popular with researchers for developing human cancer xenografts in mice and especially patient-based xenografts [9] which is the reason it was chosen to carry out the pharmacokinetic analyses. Representative HPLC chromatograms are shown in Figure 1. The pharmacokinetic profile of GEM after administration is shown in Figure 2. The values of the pharmacokinetic parameters for the SC administration of gemcitabine, Τ_1/2_, Tmax, Cmax, AUC_0–t_ and clearance (Cl) were 64.49 min, 5.00 min, 264.88 μmol/L, 9351.95 μmol/L*min and 0.0103 (mg)/(μmol/L)/min, and for IP administration, Τ_1/2_, Tmax, Cmax, AUC_0–t_ and Cl were calculated to be 59.34 min, 5.00 min, 300.73 μmol/L, 8981.35 μmol/L*min and 0.0108 (mg)/(μmol/L)/min, respectively. There were no significant differences between SC and IP administration of GEM in mice under the experimental conditions tested here (*p* > 0.05, Mann–Whitney U test, Table 3). Measurable amounts of GEM were observed up to 6 h post-drug administration.

## 4. Discussion

GEM is a cytidine analogue in which the 2′-carbon of the ribose is linked to two fluorine atoms. Is a prodrug that is converted to its active metabolites, which act by replacing cytidine during DNA replication, resulting in tumor growth arrest and induction of cell death in malignant cells. It has a broad spectrum of anti-tumor activity, with one of the most important clinical applications being its use as a single agent in the first-line treatment of patients with pancreatic cancer.

It has been proposed that the optimal plasma concentration of GEM, which maximizes the rate of GEM triphosphate formation, is approximately 20 μΜ [10,11] at a dose rate of 10 mg m^−2^ min^−1^ [12]. It has also been reported that the Cmax of GEM in patients with advanced pancreatic adenocarcinoma ranges from approximately 25 to 75 μΜ for dose regimens of 3000 to 6500 mg m^−2^ at the same dose rates [13].

However, when administered intravenously, gemcitabine is extensively metabolized by plasma and liver CDA. It has long been reported that plasma clearance of gemcitabine varies greatly between patients receiving the same dose [14,15], and it appears that patient-specific characteristics (i.e., covariates, genetic polymorphisms, etc.) may influence drug and metabolite disposition, which could affect not only the pharmacologic activity but the safety of the drug as well. More than 10% of the patients who receive GEM develop severe adverse effects, such as low white and red blood cell counts, and low platelet counts due to bone marrow suppression. Other common side effects associated with GEM administration include vomiting and nausea, difficulty breathing, rashes and itchy skin, hair loss, flu-like symptoms, edema, fever, loss of appetite (which can contribute to the development of cancer cachexia), headaches, difficulty sleeping, fatigue, and many others that significantly decrease the quality of life of patients [16]. In a case study, Mascherona et al. [17] reported the development of clinically significant acute hepatic injury after using gemcitabine in a 73-year-old man. The hepatic injury was resolved after GEM removal. Jha et al. [18] reported the development of thrombotic micro-angiopathy (TMA) manifesting as nephrotic syndrome with renal dysfunction and posterior reversible encephalopathy syndrome (PRES) in a young male with pancreatic carcinoma who received gemcitabine as adjuvant chemotherapy, with these adverse reactions withdrawing after discontinuation of the drug. Although not as common, deaths associated with GEM administration have also been reported, all related to CDA polymorphisms resulting in reduced metabolism of the drug [19,20].

As it is generally unclear how these variabilities may affect the anti-cancer activity and safety of the drug, pharmacokinetics may be useful in the context of therapeutic drug monitoring towards more rational individualized drug dosing and treatment regimens that include GEM.

Based on this need, a simple and cost-effective HPLC-UV assay for the monitoring of GEM in serum was developed and validated in the current study, and subsequently implemented preclinically in a proof-of-concept effort for the pharmacokinetic analysis of GEM in mice. Several HPLC coupled with UV detection methods (Table 4) [4,7,21,22,23,24,25,26,27], as well as mass spectrometry-based methods [28], have been developed for the determination of GEM plasma levels. While HPLC methods with MS detection show high sensitivity, fast analysis time, and small sample size, they require expensive and sophisticated instrumentation that requires highly trained personnel. On the other hand, UV-HPLC methods are more cost-effective while providing adequate limits of quantitation and satisfactory throughput. Finally, a rapid automated immunoassay for the quantification of GEM in human serum has also been developed [29]. Nevertheless, to the best of our knowledge, the manufactured reagent kit is not commercially available so far.

Our method is characterized by simple sample treatment that incorporates a single protein precipitation step, simple chromatographic conditions, and instrumental configurations. Thus, we omit problems that arise from using more complicated equipment such as LC-MS/MS, which requires advanced trained personnel and involves considerable cost relative to a simple HPLC-UV installation.

A single protein precipitation step was chosen as it was sufficient for sample treatment, conferring it an advantage over more complicated and time-consuming procedures like liquid–liquid extraction or solid-phase extraction [27,30]. The protein precipitation procedure has also been applied in several methods using either organic solvent [7,21,22,23,25] or TCA [26]. The protein precipitation with perchloric acid was used only in one other study [4]. In our study, 6% *v*/*v* perchloric acid was used as a precipitating agent since better recovery of GEM was observed during the analysis of samples in comparison with 3% and 4% *v*/*v* perchloric acid. The recovery was optimized by adding 80 μL of 6% perchloric acid thus minimizing sample dilution. The supernatants were clear, and under no circumstances was the analysis of the samples affected by the phenomenon of hemolysis.

In the present study, the isocratic elution ensured adequate separation of GEM, its main metabolite dFDU, and the IS, while the analysis time of GEM and the IS was approximately 12.5 min. More specifically, the retention time of GEM, IS, and dFDU was determined to be 9.56 ± 0.53 (*n* = 70), 12.19 ± 0.58 (*n* = 70) and 13.64 ± 0.88 (*n* = 70), respectively. In comparison with other published methods [4], the use of the ion-pair reagent was avoided, as it resulted in a slow equilibration time of the column. In parallel, it is commonly known that the presence of an ion-pair reagent can be harmful for silica; therefore, the avoidance of the mentioned reagent would be preferable [31]. GEM was analyzed under isocratic conditions in several other methods, and the reported duration of analysis was in the range of 10 to 24 min [4,7,21,23,24,25]. Therefore, the analysis time of GEM bears resemblance to the shorter reported method employing simple protein precipitation sample pretreatment [4]. While gradient elution confers a shorter elution time of GEM [9], the isocratic elution mode was preferable thanks to its simplicity, since no re-equilibration of the column is required.

During the validation of the method, several substances were tested as internal standards: 2-deoxycytidine, 1-methylxanthine, 1-methyluric acid, 7-methylxanthine, 7-methyluric acid, and 3-methylxanthine were tested, but none of these exhibited adequate separation from GEM, dFDU, and matrix interferences. Finally, the compound selected as the internal standard was 1,7U, since it exhibited satisfactory separation from GEM and dFDU with acceptable elution time. Additionally, 1,7U is an inexpensive analyte that is not included in the human diet and is not a prescribed drug.

The pharmacokinetic study was performed on male immunodeficient mice of the NOD.Cg-Prkdc^scid^/J strain, commonly referred to as NOD SCID mice, for determining GEM levels in the serum. To assess the differences between the SC and IP routes of GEM administration, two PK studies were conducted where GEM was administered either IP or SC. The IP route of drug administration is commonly used in small experimentation animals, as the intravenous (IV) method is difficult to use due to the sensitive veins, restraint, and handling of mice. Although the IP route is considered the routine mode, it has important drawbacks and risks that could affect the welfare of the mice during the experiments. These limitations include pain, stress, discomfort, and hemorrhage. Moreover, another phenomenon is the sensitivity of the peritoneal cavity, especially in the case of irritating substances and solutions of which the pH is not physiologic. In addition, since a significant number of experimental assays require repeated drug administration under aseptic conditions, there is an increased likelihood of needle penetration into the muscle, subcutaneous tissue, colon, and other abdominal organs, which may affect the animal’s health and/or result in death. Thus, the current route requires sophisticated and well-trained operators [32]. On the other hand, the SC route is a simple form of drug administration characterized by a low risk of systemic infection, less pain, and ease of performance in mice, avoiding the limitations of the IP route. It also provides the possibility of divergent injection sites in the case of multiple dosing [32,33]. In the present study, five major PK parameters, i.e., Τ_1/2_, Tmax, Cmax, AUC_0–t_, and Cl, were assessed using PKSolver add-in [8], and no significant differences were observed between the two routes of GEM administration, indicating that the SC route could be an alternative mode of GEM administration.

The current method compared to most of the available methods is characterized by more favorable features. Low cost, a simple sample preparation procedure, linear responses in the concentration range of 1 to 400 μM of GEM, and fast analysis (12.5 min) with the retention time of the GEM being around 10 min are among its main characteristics. It is also characterized by quite a low limit of detection (0.166 ± 0.022 μΜ), while it can also clearly detect the major metabolite of GEM, dFDU. These characteristics make the HLPC-UV method developed and described here attractive for use in clinics. Thus, in this context, we further tested its applicability in commercially available human serum samples. The chromatographic analysis revealed no interferences at the retention times of GEM and IS, suggesting that the method might potentially be used in clinical practice for monitoring GEM levels in the serum of cancer patients.

## 5. Conclusions

In conclusion, a simple, sensitive, specific, rapid, and inexpensive reversed-phase HPLC method coupled with UV detection was developed and validated for the detection and quantification of GEM in mouse and human serum. Successful implementation of the method in a proof-of-concept mouse model PK study suggests that the current method could be an important tool for drug monitoring and studying the pharmacokinetics of GEM, at least in a preclinical setting. As it has not yet been tested in patient samples and given its favorable characteristics, studies are ongoing in our laboratory to explore the potential of this method to be equally useful for monitoring GEM levels in human serum samples in the context of individualized treatment of cancer patients receiving the drug.

## Figures and Tables

**Table 1:** Data on the precision and accuracy of GEM calibrators. Percentages of CV and BIAS show the mean of independent replicates, *n* = 2.

Calibrator Concentration (μΜ)	Mean Measured Concentration (μΜ)	CV (%)	Bias (%)
1	1.06	0.31	6.43
10	9.63	1.97	−3.66
50	48.23	0.13	−3.54
100	100.60	1.72	0.60
200	208.34	1.52	4.17
400	401.96	2.91	0.49

**Table 2:** Intra-day and inter–day precision and accuracy for GEM quality controls (QC) generated in normal mouse serum, *n* = 5.

INTRA-DAY	MEAN	SD	CV (%)	BIAS (%)
QC-3 μΜ	3.50	0.17	4.89	16.55
QC-25 μΜ	27.81	2.05	5.98	11.25
QC-125 μM	126.84	3.70	2.92	1.47
QC-375 μΜ	347.81	26.16	7.52	−7.25
**INTER-DAY**	**MEAN**	**SD**	**CV (%)**	**BIAS (%)**
QC-3 μΜ	3.44	0.33	9.56	14.70
QC-25 μΜ	26.05	1.61	6.16	4.21
QC-125 μM	126.01	4.51	3.58	0.80
QC-375 μΜ	366.87	14.69	4.00	−2.17

**Table 3:** The pharmacokinetic parameters (median, (range)) after IP and SC administration of gemcitabine in mice are shown, *n* = 5. AUC; area under curve. Cl; clearance. * Statistical analysis was performed by Mann–Whitney *U* test.

Parameter (Unit)	IP	SC	*p* *
T_1/2_ (min)	59.34 (45.89–134.52)	64.49 (55.13–69.43)	1.00
Tmax (min)	5.00 (5.00–5.00)	5.00 (5.00–5.00)	1.00
Cmax (μmol/L)	300.73 (279.17–305.66)	264.88 (234.37–339.90)	0.31
AUC_0–t_ (μmol/L*min)	8981.35 (7735.79–9354.28)	9351.95 (7811.82–9851.25)	0.31
Cl (mg)/(μmol/L)/min	0.0108 (0.0103–0.0125)	0.0103 (0.0098–0.0120)	0.15

**Table 4:** The published HPLC-UV methods for the determination of GEM in serum/plasma are summarized in this table. The proposed method is the method developed and described in the current work.

Reference	Column	Elution Mode	Mobile Phase	Sample Matrix	Sample Pre-Treatment	Internal Standard	Sample Volume	Analysis Time	Analytical Range (μΜ)	LLOQ	LLOD
[4]	C18	Isocratic	10% *v*/*v* acetonitrile/90% *v*/*v* sodium phosphate	Human plasma	protein precipitation (perchloric acid)	2′-Deoxycytidine	250 μL	10 min	2–100	2 μM	0.02 μΜ
[7]	C18	Gradient	0.3–3% *v*/*v* acetonitrile /phosphate buffer	Human plasma & Rat serum	protein precipitation (acetonitrile)	2′-Deoxyuridine	200 μL	7 min	0.076–75.99	0.076 μΜ	0.038 μΜ
[21]	NH2 (Amino)	Isocratic	630 mL cyclohexane, 150 mL 1,2-dichloroethane, 220 mL methanol, 1 mL purified water, 0.5 mL glacial acetic acid, 1 mL triethylamine.	Human plasma	protein precipitation (isopropanol—ethyl acetate)	2′-Deoxycytidine	200 μL	15 min	0.19–9.1	0.19 μΜ	-
[22]	C18	Gradient	Solvent A: 98% *v*/*v* sodium acetate/2% *v*/*v* methanol—Solvent B: 90% *v*/*v* sodioum acetate/10% *v*/*v* methanol	Human plasma	protein precipitation (acetic acid)	2′-Fluorodeoxycytidine (FdC)	500 μL	17.5 min	0.5–150	-	-
[23]	NH2 (Amino)	Isocratic	30% *v*/*v* methanol/50% *v*/*v* cyclohexane/20% *v*/*v* 1,2-dichloroethane	Human plasma	protein precipitation (isopropanol)	-	200 μL	10 min	0.76–189.97	0.57 μΜ	0.38 μΜ
[24]	C18	Isocratic (ion pair)	10 mM sodium 1-heptanesulfonate in ammonioum dihydrogen phosphate buffer solution (20 mM, pH 3.1): methanol (83:17% *v*/*v*)	Human plasma	protein precipitation (trichloroacetic acid)	-	100 μL	24 min	0.3–75.99	0.3 μΜ	0.19 μΜ
[25]	C18	Isocratic	Acetate ammonium buffer solution (pH 5.5)—acetonitrile (97.5:2.5% *v*/*v*)	Human plasma	protein precipitation (methanol-acetonitrile 1:9 *v*/*v*)	Floxuridine	900 μL	18 min	0.76–37.98	0.76 μΜ	0.38 μΜ
[26]	C18	Gradient	Solvent A: Sodium acetate buffer pH = 5 Solvent B: acetonitrile. %A:B%: 98.5:1.5 (*v*/*v*)	Human plasma	protein precipitation (methanol)	Cytarabine crystalline (4-amino-1-β-D-arabinofuranosyl-2(1H)-pyrimidinone	200 μL	13 min	00.98–37.99	0.95 μΜ	0.84 μΜ
[27]	HILIC-Amide	Isocratic	90% *v*/*v* acetonitrile/10% *v*/*v* ammonioum acetate	Human plasma	liquid—liquid extraction	Cytarabine	190 μL	8.5 min	1.9–189.97	-	-
Proposed method	C18	Isocratic	3% *v*/*v* methanol/97% *v*/*v* phosphate buffer solution	Mouse serum	protein precipitation (perchloric acid)	1,7-Dimethyluric Acid (1,7U)	200 μL	12.5 min	1–400	1 μΜ	0.17 μΜ

## Data Availability

Data are available upon request to the corresponding author.
